# Investigational Drug Treatments for Triple-Negative Breast Cancer

**DOI:** 10.3390/jpm11070652

**Published:** 2021-07-10

**Authors:** Christos Damaskos, Nikolaos Garmpis, Anna Garmpi, Konstantinos Nikolettos, Panagiotis Sarantis, Vasiliki E. Georgakopoulou, Afroditi Nonni, Dimitrios Schizas, Efstathios A. Antoniou, Michalis V. Karamouzis, Nikos Nikolettos, Konstantinos Kontzoglou, Alexandros Patsouras, Errika Voutyritsa, Athanasios Syllaios, Evangelos Koustas, Nikolaos Trakas, Dimitrios Dimitroulis

**Affiliations:** 1 Renal Transplantation Unit, Laiko General Hospital, 11527 Athens, Greece; 2 N.S. Christeas Laboratory of Experimental Surgery and Surgical Research, Medical School, National and Kapodistrian University of Athens, 11527 Athens, Greece; nikosg22@hotmail.com (N.G.); k.nikolettos@yahoo.gr (K.N.); efstathios.antoniou@gmail.com (E.A.A.); kckont@med.uoa.gr (K.K.); patsouras.alexandros@gmail.com (A.P.); errikav@hotmail.gr (E.V.); 3 Second Department of Propedeutic Surgery, Laiko General Hospital, Medical School, National and Kapodistrian University of Athens, 11527 Athens, Greece; dimitroulisdimitrios@yahoo.com; 4 First Department of Propedeutic Internal Medicine, Laiko General Hospital, Medical School, National and Kapodistrian University of Athens, 11527 Athens, Greece; annagar@windowslive.com; 5 Molecular Oncology Unit, Department of Biological Chemistry, Medical School, National and Kapodistrian University of Athens, 11527 Athens, Greece; psarantis@med.uoa.gr (P.S.); mkaramouz@med.uoa.gr (M.V.K.); vang.koustas@gmail.com (E.K.); 6 Department of Pulmonology, Laiko General Hospital, 11527 Athens, Greece; vaso_georgakopoulou@hotmail.com; 7 First Department of Pathology, Medical School, National and Kapodistrian University of Athens, 11527 Athens, Greece; afnonni@med.uoa.gr; 8 First Department of Surgery, Laiko General Hospital, Medical School, National and Kapodistrian University of Athens, 11527 Athens, Greece; schizasad@gmail.com (D.S.); nh_reas@hotmail.com (A.S.); 9 Obstetric-Gynecologic Clinic, Medical School, Democritus University of Thrace, 68100 Alexandroupolis, Greece; nnikolet@med.duth.gr; 10 Department of Biochemistry, Sismanogleio Hospital, 15126 Athens, Greece; nitrakas@otenet.gr

**Keywords:** novel therapeutic strategies, immunotherapy, targeted therapies, PI3kb/mTOR inhibitors, PARP inhibitors, histone deacetylase inhibitors

## Abstract

Triple-negative breast cancer (TNBC) is an aggressive subtype of breast cancer (BC) and accounts for 10–20% of cases. Due to the lack of expression of several receptors, hormone therapy is largely ineffective for treatment purposes. Nevertheless, TNBC often responds very well to chemotherapy, which constitutes the most often recommended treatment. New beneficial targeted therapies are important to be investigated in order to achieve enhanced outcomes in patients with TNBC. This review will focus on recent therapeutic innovations for TNBC, focusing on various inhibitors such as phosphoinositide 3-kinase (PI3K) pathway inhibitors, poly-ADP-ribosyl polymerase (PARP) inhibitors, aurora kinase inhibitors, histone deacetylase inhibitors (HDACIs), and immune checkpoint inhibitors.

## 1. Introduction

Breast cancer (BC) is considered the second most commonly occurring pathology in the world [1]. BC is more frequently diagnosed in less developed and industrialized countries, it also constitutes the second notable cause of mortality in Europe and the United States after lung cancer [1,2]. Additionally, according to the American Cancer Society, about 12% of women in the USA are prone to develop BC during their lifetime [3,4,5,6].

**Figure 1:**
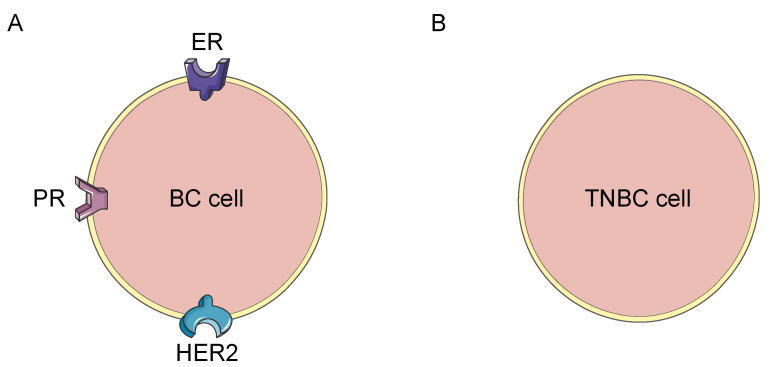
The lack of expression of receptors in triple-negative breast cancer. (**A**): Most breast cancer cells express estrogen receptor, progesterone receptor and human epidermal growth factor receptor 2. (**B**): Triple-negative breast cancer lacks the expression of these receptors. BC: Breast cancer; ER: Estrogen receptor; PR: Progesterone receptor; HER2: Human epidermal growth factor receptor 2; TNBC: Triple-negative breast cancer.

Triple-negative breast cancer (TNBC) is a less common type of BC. About 10–20% of BCs are TNBC. TNBC consists of cancer cells, which either do not express estrogen and progesterone receptors or produce the protein named HER2 (Figure 1). These cancers tend to be more common in women younger than the age of 40, who are usually African American [7].

Risk factors for TNBC, are not clear. Human genes BRCA1 and BRCA2 produce tumor suppressor proteins. These proteins participate in damaged DNA repairing and, therefore, play a crucial role in ensuring the stability of each cell’s genetic material. Since these genes are mutated, DNA damage may not be repaired properly. As a result, cells are more likely to develop additional genetic alterations, which may lead to cancer. Such gene mutations are the inherited mutations in BRCA1 and BRCA2 genes, which are reported to increase the risk of female TNBC [8,9].

Treatment for TNBC depends on different factors, such as the stage and the grade of the cancer. It is usually a combination of surgery, radiotherapy and chemotherapy. Unlike most other types of BC, TNBC does not express estrogen, progesterone and HER2 receptors. Therefore, hormone therapy is largely ineffective for treatment purposes. Nevertheless, TNBC often responds very well to chemotherapy [10,11,12]. However, chemotherapy can cause various serious adverse effects such as cardiotoxicity, myelosuppression, alopecia and gastrointestinal problems [13]. Neutropenia and neutropenic fiber can be fatal for the patients [14]. Except for toxicity, drug resistance to chemotherapy is another major problem. MicroRna-based therapies have been also examined in animal models with BC, but more research needs to be done [15]. Thus, it is of paramount importance to create new drugs, personalized to the type of cancer and the needs of the patient, in order to increase efficacy and reduce toxicity.

Major effort has been devoted by researchers in order to classify TNBC. Technology has facilitated researchers to analyze numerous data to compare different TNBCs and classify them in subgroups based on their similarities. Several ways of categorization of TNBCs have been reported such as the molecular classification, the immune classification, the classification based on differential prognosis, based on the cell type ambulating in the tumor environment, based on the presence or absence of androgen receptors or based on cellular type [16].

Given that the progression of cancer is often controlled via epigenetic processes, there is a growing interest in research focusing on mechanisms, genes and signaling pathways related to carcinogenesis with epigenetic modulation of gene expression. For example, histone deacetylases (HDACs) have a significant impact on chromatin remodeling and epigenetics. Therefore, their inhibitors consist of an appealing field for targeted therapy against BC and are widely studied [17,18]].

Along the same line with HDACs, numerous studies and both clinical and laboratory trials are taking place, in order to provide new targets and improve prognosis for TNBC. PARP inhibitors, the PI3K/AMT/TOR pathway, anti-angiogenetic factors, as well as immunotherapy are potential targets for the treatment of TNBC. This current review presents up-to-date studies, focusing on the progress made in the field of targeted therapies for TNBC.

## 2. Material and Methods

**Figure 2:**
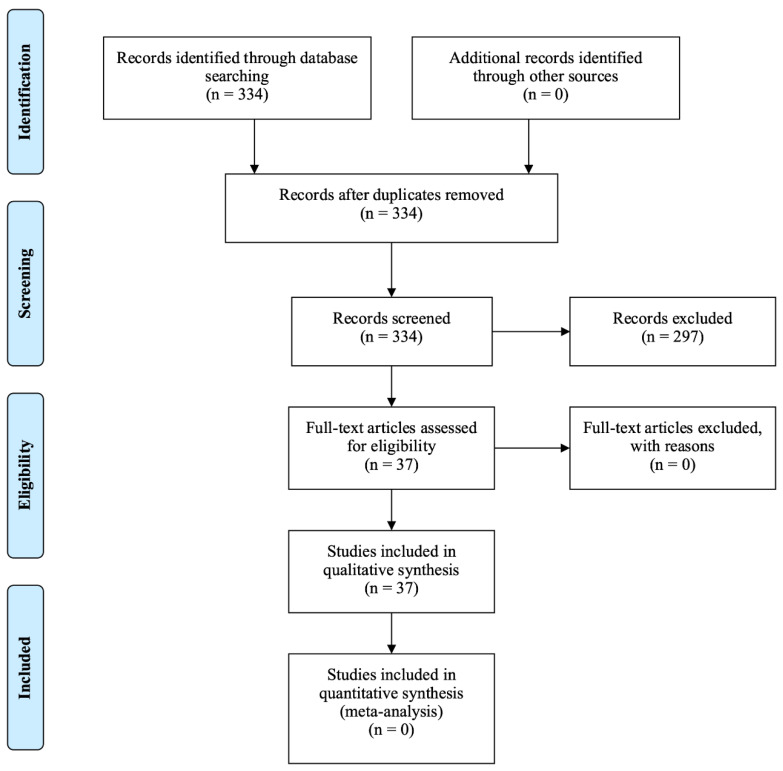
PRISMA flow diagram for the current literature review.

A literature search was conducted in the MEDLINE (via PubMed) library in order to retrieve articles focusing on TNBC. The search strategy was based on the use of keywords such as triple-negative breast cancer, clinical, laboratory trials, targeted therapies, novel therapeutic strategies, immunotherapy, PARP inhibitors, histone deacetylase inhibitors, aurora kinase inhibitors, PI3kb/mTOR inhibitors and immune checkpoint inhibitors. The search strategy included this combination (((triple-negative breast cancer) AND (clinical or laboratory trials or preclinical trials or in vitro study or in vivo study)) AND (novel therapeutic strategies or targeted therapies)) AND (PARP inhibitors or histone deacetylase inhibitors or aurora kinase inhibitors or PI3kb/mTOR inhibitors or immune checkpoint inhibitors). The PRISMA approach was used for the selection of the publications included in the review. A total of 334 records were identified. No duplicate was removed. These were screened and 297 were excluded because they did not include clinical or laboratory trials, were mainly abstracts or were written in a non-English language. Other studies were excluded because they referred to other types of cancer generally or BC and did not refer to TNBC specifically. Clinical, in vivo and in vitro studies, examining the above agents in TNBC cells were included in the review. The full-text articles assessed for eligibility numbered 37 and none of them were excluded. The inclusion process is presented in Figure 2.

## 3. Results

### 3.1. PI3K/AKT/mTOR Pathway

**Figure 3:**
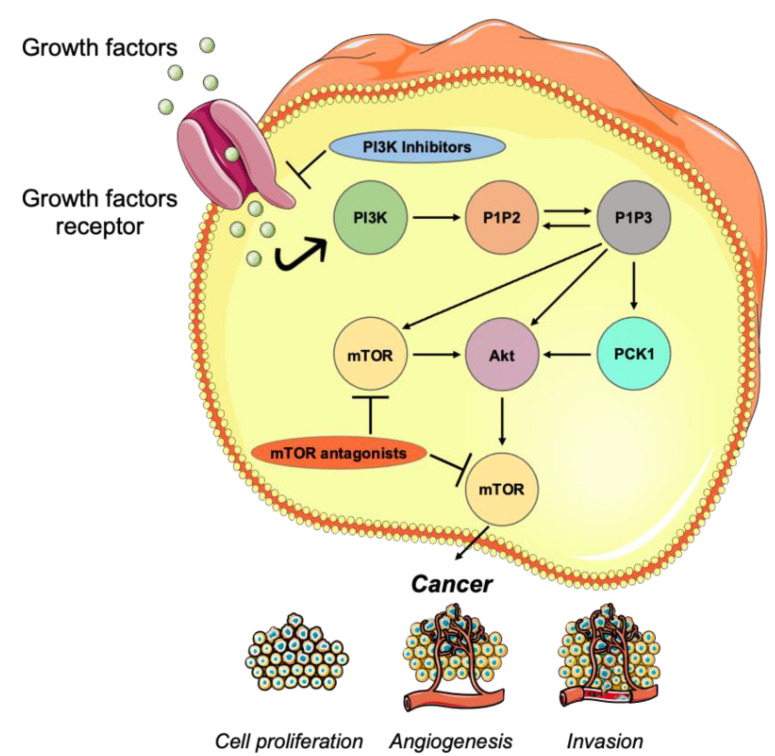
PI3K/Akt/mTOR pathway as a therapeutic target against triple-negative breast cancer.

The fallible regulation of mTOR signaling and especially phosphoinositide-3-kinase PI3K/Akt/mTOR pathway is considered to be related to malignancy [19,20]. The mTOR pathway is altered in patients with TNBC, thus it is responsible for aggressive tissue invasion. PIK3CA gene activating mutations represent common mutations, estimated at 20%. Phosphorylation reactions, which are taking place due to the PI3K/Akt/mTOR pathway, lead to cancer cell growth, cell proliferation and angiogenesis via the depletion of inositol polyphosphate 5-phosphatase PIPP and activation of serine/threonine kinase AKT. Additionally, over-expression of regulators, such as epidermal growth factor receptor (EGFR), protein kinases, such as Akt, or when the mutations occur, are reported to be correlated to tumor metastasis and invasion, with the production of matrix metalloproteinase 2 and degradation of collagen type IV (Figure 3) [21,22,23,24].

In 2014, Ganesan et al. conducted a phase I trial, in which 98 consecutive patients with advanced or metastatic TNBC participated [25]. 12 of 98 enrolled patients had complete response (one patient), partial response (seven patients) or stable disease (four patients) for at least 6 months. Patients received matched therapy (chemotherapy and targeted therapy) compared with those to non-matched (either targeted agents alone or chemotherapy alone) showed improved results and longer progression-free survival. Among the 12 patients who had stable disease for at least 6 months, complete response and partial response, five were treated with the same combination of chemotherapy receiving liposomal doxorubicin, with anti-angiogenic therapy receiving bevacizumab and with mTOR inhibitor therapy receiving temsirolimus. Three of these Five patients presented metaplastic histology. Among 43 patients evaluated for alterations in the PI3K/AKT/mTOR pathway, 21 presented at least one alteration (including mutations in PIK3CA, PIK3R1, PTEN, NF2, deletion in PTEN, PIK3CA amplification and PTEN loss on IHC). 16 of these 21 patients received therapies with at least one drug that targets the PI3K/AKT/mTOR pathway and 15 were evaluable for response. This study suggests that patients with metastatic TNBC, treated with combinations of chemotherapy and angiogenesis and/or PI3K/AKT/mTOR inhibitors presented improved results.

In 2016, Basho et al. conducted a phase I trial, in which 52 women with metaplastic TNBC participated for 21 days [26]. These 52 women were treated with liposomal doxorubicin, bevacizumab and temsirolimus or liposomal doxorubicin, bevacizumab and everolimus. In 32 patients, the examination of breast tissue revealed a PI3K aberration. These patients had a better objective response rate with the use of mTOR inhibitors. Concerning the patient response rate, it was 21% (complete response 8%, partial response 13%) and 10 patients had stable disease for at least 6 months. As a result, the presence of PI3K pathway aberration was related to a significant improvement in patient response rate (31% vs. 0%).

In 2018, Basho et al. conducted a phase I trial, in which 43 patients with non-metaplastic TNBC and 59 patients with advanced metaplastic BC participated for a period of 5 years [27]. During this study, mTOR inhibition, temsirolimus or everolimus, with liposomal doxorubicin and bevacizumab were used. Average progression-free survival for patients with non-metaplastic TNBC and patients with metaplastic BC was 2.5 months and 4.8 months, respectively. Median overall survival for patients with non-metaplastic TNBC and patients with metaplastic BC was 3.7 months and 10 months, respectively. On the basis of these data, treatment with mTOR inhibition, temsirolimus or everolimus, with liposomal doxorubicin and bevacizumab appeared to be more effective in metaplastic BC compared with TNBC.

In 2019, Lee et al. conducted a clinical trial in which they tested the combination of everolimus and eribulin in patients with metastatic TNBC [28]. They used various dosages of the above medications in order to examine both the efficacy and complications. Among the 25 patients, nine were stable, nine reported partial response and seven had progressive disease. Toxicity due to chemotherapy included hematological disorders, fatigue, stomatitis, and hyperglycemia. Median overall survival was 8.3 months and median time for progression of the disease was 2.6 months. The above regimen showed safety and modest efficacy.

In 2020, Owusu-Brackett et al. reported a study, in which in vitro cell viability assay and immunoblotting indicated that PTEN loss was related to AZD8186 sensitivity in TNBC [29]. Colony formation assay was also studied and confirmed the sensitivity of PTEN deficient cell lines to AZD8186. AZD8186, being an inhibitor for the PI3K signaling in PTEN loss in TNBC, was evaluated as therapy in combination with paclitaxel and eribulin. The synergistic effects of these drugs led to the growth inhibition in PTEN loss cells. AZD8186 initiated apoptosis in PTEN loss cells when it was used in combination with paclitaxel. Moreover, in vivo, AZD8186 had limited activity when it was used as the only agent, but it resulted in an advanced anticancer activity when it was combined with paclitaxel in MDA-MB-436 and MDA-MB-468 cell-line xenografts. Finally, AZD8186 improved the anticancer activity of anti-PD1 antibodies in the PTEN-deficient BP murine melanoma xenograft model, but it did not lead to improved results in the PTEN-wild-type CT26 xenograft model.

In 2021, Ma et al. conducted a study in which they used cell lines such as MDA-MB-231, A549 and HeLa cell lines [30]. They tested the actions of anilide in the down-regulation of PI3K/Akt/mTOR signaling pathway. They showed that anilide can enhance apoptosis and inhibit migration and proliferation of TNBC cells.

### 3.2. PARP Inhibitors

**Figure 4:**
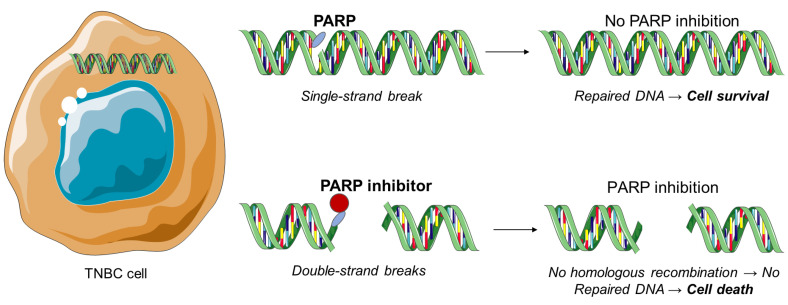
PARP inhibition as a therapeutic strategy against triple-negative breast cancer.

The polyadenosine diphosphate-ribose polymerase, also called poly (ADP-ribose) polymerase or PARP, is a group of various proteins that participate in molecular mechanisms, leading to recovery of the cells from DNA damage (Figure 4) [31]. PARP inhibitors constitute the most important therapeutic drugs for the BRCA-1 and BRCA-2 mutations and therefore against TNBC. Exposure to chemotherapy results in PARP expression in TNBC. Moreover, PARP-1 and PARP-2 proteins are associated with DNA repair processes by repairing proteins and binding to DNA breaks [32,33,34,35,36,37]. Trapped PARP-DNA complexes are extremely cytotoxic and present high anti-proliferative and anticancer activity [38].

In 2015, Llombart-Cussac et al. conducted a Phase II trial, in which 141 patients with TNBC Stage II-IIIa were randomized to receive paclitaxel alone (PTX) or in combination with iniparib, either once a week (PWI) or twice a week (PTI) for 12 weeks [39]. The initial target was the pathologic complete response (pCR) in the breast. Notably, pCR rate was similar among the three arms (21, 22, and 19% for PTX, PWI, and PTI, respectively). No significant differences were observed in serious side effects leading to the termination of the treatment among the three arms. When iniparib was added to PTX, it did not provide any enhanced antitumor activity or toxicity. According to these results, further evaluation of the combined treatment with iniparib at these doses and paclitaxel in TNBC is not suggested. Finally, but not least important, it should be noted that iniparib does not exert inhibition against PARP in vitro [40].

In 2016, Kummar et al. presented a phase II study, in which 45 patients with TNBC were randomized to be treated with oral cyclophosphamide with or without oral veliparib in 21-day cycles [41]. More specifically, patients who received cyclophosphamide and patients who received the combination of drugs were compared focusing on disease progression. 18 patients were treated with cyclophosphamide alone and 21 with the combination of drugs. Lymphopenia was the most common toxicity observed in both groups. Concerning the response rates and progression free survival, they did not present any notable differences between both treatment groups. As a result, the addition of veliparib to cyclophosphamide, at the dose and schedule evaluated, did not lead to any improved results for the treatment in patients with TNBC.

In 2017, Evans et al. reported a study, in which patient-derived xenografts (PDXs) were obtained from surgical samples of recurrent tumors [42]. During this study, 26 PDXs were developed from 25 patients. 22 derived patients with residual disease treated with neoadjuvant chemotherapy, and 24 derived from patients with TNBC. The 26 PDXs provided a heterogeneous set of mutations and were all related to TNBC. Concerning RPPA, PDXs activated in a different way the PI3K and MAPK and presented different sensitivity to chemotherapy. On the contrary to PI3K, mTOR, and MEK inhibitors that initiate growth but not tumor regression, the PARP inhibitor talazoparib led to significant regression in 5 of 12 PDXs. On the basis of these data, PARP inhibition can have notable activity, causing regression in various molecular subtypes and PDXs are potential predictive biomarkers in targeted therapies.

In 2020, Pothuri et al., used the combination of veliparib and doxorubicin in patients with TNBC [43]. Drugs were administrated in various dosages. Although complete clinical response was observed in two cases, and the anti-tumor efficacy was generally acceptable, complications such as oral squamous cell carcinomas appeared.

In 2021, Eikesdal et al. conducted a clinical trial in which they tested olaparib in TNBC patients, without previous chemotherapy exposure [44]. DNA sequencing and methylation analysis of the tumor cells were conducted before and after the administration of olaparib. They demonstrated that olaparib is effective against treatment-naïve TNBC cells with HR deficiency.

### 3.3. Aurora Kinase Inhibitors

Aurora kinases constitute cell cycle-regulated serine/threonine kinases and are reported to be important for mitosis [45,46,47]. In humans, the Aurora kinases are categorized in three groups, including Aurora-A, Aurora-B, and Aurora-C, which each share a conserved C-terminal catalytic domain but differ in various points, such as their sub-cellular localization, substrate specificity, and function during mitosis. Their deregulation leads to G2-M arrest, apoptosis and ceases mitosis [48]. Moreover, over-expression of Aurora-A and Aurora-B has been proved to lead to a wide variety of tumors as it transforms epithelial cells to mesenchymal ones and offers them abilities of stem-like cells [49,50,51,52,53,54,55,56]. As a result, developing Aurora kinase inhibitors, as anti-cancer drugs, has attracted academic attention.

In 2014, Huck et al. reported a study in which MLN8237, also known as alisertib, being a selective Aurora A inhibitor, was evaluated as an anticancer drug in multiple solid tumors [57]. Alisertib was in combination with docetaxel or paclitaxel was estimated in in vivo models of TNBC, focusing on the anticancer activity. When alisertib was combined with taxanes, an additive, and synergistic anticancer activity was observed. When multiple dose levels of alisertib and paclitaxel were used, tumor growth inhibition was achieved. Patients who received the highest dose of alisertib being or not being combined with 60 or 80 mg of paclitaxel, presented similar results. As a result, these observations can be used in order to optimize the combination therapies using other therapeutic agents.

In 2018, Carducci et al. conducted a trial, in which patients with TNBC were treated with dose-escalation and dose-expansion phases with AMG 900, a pan-Aurora kinase inhibitor [58]. Dose expansion investigated focusing on three tumor types: taxane- and platinum-resistant ovarian cancer, taxane-resistant TNBC and castration-resistant and taxane- or cisplatin/etoposide-resistant prostate cancer. AMG 900 presented rapid absorption in once-daily dosing. The maximum tolerated dose was 25 mg/day, increasing to 40 mg/day with granulocyte colony-stimulating factor. The treatment-related adverse effects that were observed were neutropenia, anemia, leukopenia, and thrombocytopenia. When the dose was expanded 3 of 29 enrolled patients with ovarian cancer presented partial response, while the median duration of response was 24.1 weeks. Five of nine patients found positive for p53 expression experienced well response to treatment. On the other hand, no response was observed in patients with TNBC. Similar results, with minimal clinical response, and serious adverse effects were observed in a clinical trial conducted by Tolcher et al. [59]. Specifically, they used trametinib and uprosertib in patients with TNBC or melanoma. The anti-tumor efficacy was minimal, whereas adverse effects such as severe diarrheas or rashes appeared.

### 3.4. Histone Deacetylase Inhibitors

**Figure 5:**
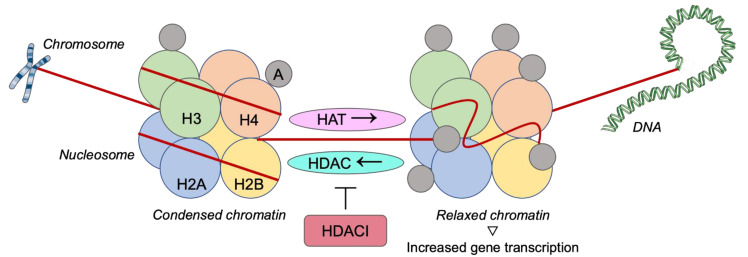
Histone deacetylases inhibition as a therapeutic strategy against triple-negative breast cancer. H: Histone; A: Acetylgroup; HAT: Histone acetyltransferase; HDAC: Histone deacetylase; HDACI: Histone deacetylase inhibitor.

A wide range of histone deacetylase inhibitors (HDACIs) have been isolated from natural sources or have been developed in laboratory in order to be tested in clinical studies [60]. HDACIs participate in various mechanisms including the chromatin remodeling via deacetylation of histones that prevents gene transcription, the DNA target that leading to DNA damage though a mechanism of oxidative stress, or the participation in pathways of apoptosis through the up-regulation of proapoptotic proteins and down-regulation of antiapoptotic proteins. Moreover, it has been reported that HDACIs have an anti-angiogenic effect, decrease the expression of vascular endothelial growth factor (VEGF) receptor and prevent proliferation, invasion, and migration of endothelial cells [61,62,63]. It has also been proved that numerous HDACIs have an impact on the immune system functions [64]. Participating in these mechanisms, HDACIs would be potential agents in cancer therapy, especially in combination with targeted agents (Figure 5).

In 2015, Min et al. reported in vivo and in vitro studies, in which the potential of suberoylanilide hydroxamic acid (SAHA), an HDACI, to improve the anti-tumor effects of olaparib on TNBC cell lines was investigated [65]. More specifically, the effect of SAHA on the expression of HRR-associated genes was studied. The in vitro results were confirmed in vivo utilizing a human BC xenograft model. As a result, the combination of olaparib and SAHA inhibited efficiently the growth of TNBC cells. This outcome was related to down-regulation of the proliferative signaling pathway, increased apoptotic and autophagic cell death, and accumulation of DNA damage.

In 2018, Ono et al. conducted a study, in which the synergistic effect of OBP-801, a HDACI, and eribulin in TNBC cell lines was evaluated [66]. Flow cytometry analysis was conducted to investigate the treatment of cell lines with the combination of OBP-801 and eribulib and the induction of apoptosis. According to the experimental findings the combination OBP-801 with eribulin presented a synergistic inhibition of the growth in TNBC cells, as well as the enhancement of apoptosis. Moreover, it was proved that eribulin up-regulated survivin and that OBP-801 suppressed the up-regulation of survivin by eribulin. As a result, the combination of these two inhibitors provides a meaningful strategy for treating TNBC patients.

The same year, Song et al. reported a study in which the inhibition of TNBC by trichostatin A (TSA), an HDACI, was investigated [67]. The experimental findings indicated that TSA treatment results in decreased expression of CYCLIN D1, CDK4, CDK6, and BCL-XL, but increased P21 expression. Additionally, treatment with TSA in combination with doxorubicin results in inhibition of proliferation of HCC1806 and HCC38 cells. Therefore, the TSA and its combination with doxorubicin constitute promising therapeutic strategy in the therapy of TNBC.

Maiti et al. presented a study in which the effect of entinostat in the expression of anti-angiogenic and tumor suppressor genes was investigated in TNBC cells [68]. The experimental results revealed that treatment of the TNBC cells with entinostat led to the re-expression of the anti-angiogenic genes and the tumor suppressor genes. It was also found that TNBC cells with entinostat led to down-regulation of the expression of VEGF A (VEGF-A). Based on these data, HDACs may be a promising therapeutic tool for TNBC.

In 2020, Milazzo et al. conducted a study, in which the biological activity of a new antibody drug conjugate (ADC), ST8176AA1, derived from trastuzumab, which was partially reduced with tris [2-carboxyethyl] phosphine (TCEP) and ST7464AA1, the active form of the prodrug HDACI ST7612AA1 was evaluated in vitro and in vivo [69]. Enhanced anti-tumor activity of ST8176AA1 compared to trastuzumab was presented in vitro in tumor cell lines. Moreover, increased expression of ErbB2 and estrogen receptor was revealed in TNBC cells. In compliance with in vitro data, ST8176AA1 proved to have higher tumor growth inhibition than trastuzumab when tested to xenograft models of ovary and colon carcinoma, as well as in 2 patient-derived xenograft (PDX) models of pancreatic carcinoma. As a result, ST8176AA1 can be consider as an attractive novel therapeutic tool that it is worth more investigation.

### 3.5. Other Inhibitors

Alternative targeted therapies inhibiting nucleo-cytoplasmic transport have been reported [70,71]. Chromosome region maintenance 1 (CRM1), also known as exportin 1 (XPO1), is a protein transporter associated with nucleo-cytoplasmic shuttling of numerous tumor suppressor proteins (TSP) and growth regulatory factors. XPO1 is also reported to be up-regulated in many malignancies and is related to a poor prognosis [72,73,74]. In 2015, Arango et al. reported a study in which 26 BC cell lines of various cancer subtypes were evaluated, being treated with Selinexor in vitro [75]. According to this study, selinexor provided growth inhibition in all the cell lines tested. In multiple TNBC cell lines, selinexor showed a synergistic activity along with paclitaxel, carboplatin, eribulin, and doxorubicin in vitro. When selinexor was used without additional drugs, it managed to reduce tumor growth in vivo in four of five cell lines tested. As a result, selinexor shows potential therapeutic activity and could be further investigated as a treatment for TNBC.

Cyclin-dependent kinases (CDKs) complexes are known to regulate the progression of cells via the cell cycle and cycle division [76,77]. Moreover, deregulation in the cell cycle is of paramount importance in the development of cancer. CDK1 and CDK2 inhibitors constitute potential therapeutic targets concerning the TNBC [78,79,80]. In 2015, Mitri et al., conducted a phase I study, in which the maximum tolerance dose of dinacinib combined with epirubicin in patients with TNBC was determined [81]. Groups of at least two patients were treated with increasing doses of dinaciclib given on the first day followed by standard dose of epirubicin given on the second day of a 21-day cycle. For 1 year, nine patients were evaluated. Dose escalation proved to be toxic and did not apply to the second group. The first dose level also proved to be too toxic. As no treatment responses were observed, the combination of dinaciclib and epirubicin does not appear to be an effective treatment option for TNBC.

MET is a receptor tyrosine kinase that activates a variety of different cellular signaling pathways, including those associated with proliferation, migration, and invasion. Despite the fact that MET is known for participating in the control of tissue homeostasis it has also been reported to be activated in human cancers via mutation or protein over-expression [82]. In 2015, Tolaney et al. conducted a phase II study, in which tivantinib, an oral agent that targets MET, was evaluated as treatment for patients with TNBC [83]. During the study, 22 patients were enrolled. The overall response rate was 5% and the 6-month progression-free survival (PFS) was 5%, with one patient managing to achieve a partial response. The toxicity was trivial. Therefore, tivantinib although proved to be tolerated it did not achieved prespecified statistical targets for efficacy.

VEGF is a family of proteins including VEGF-B, VEGF-C, VEGF-D, and VEGF-E. VEGF family members are important in physiological angiogenic processes, including pathological conditions such as cancer [84]. VEGF inhibitors have been shown to regulate endothelial cell proliferation, migration, and survival, having potential anti-tumor activity [85]. In 2016, Pham et al. reported a study in which bevacizumab, a VEGF-pathway targeting anti-angiogenic drug, was evaluated for TNBC [86]. More specifically, bevacizumab and CRLX101, an investigational nanoparticle-drug conjugate that contains camptothecin, was tested in preclinical mouse models of orthotopic primary TNBC xenografts. Long-term efficacy of CRLX101 and bevacizumab were also tested in order to treat postsurgical, advanced metastatic BC in mice. According to this study, CRLX101 not only alone, but also combined with bevacizumab, was highly efficient, resulting in complete tumor regressions, reduced metastasis, as well as extended survival of mice with metastatic tumors. Based on these data, CRLX101 along with bevacizumab is a potential anti-angiogenic therapy for TNBC.

The epidermal growth factor receptor, also known as EGFR regulates various cellular processes, such as proliferation, differentiation, and survival. Overexpression of EGFR leads to poor outcome and carcinogenesis, including cell growth and invasion, angiogenesis, and metastasis [87,88,89,90]. Several EGFR inhibitors have been tested as potential therapeutic agents against cancer. In 2016, Brinkman et al. reported a study in which the in vivo efficiency of a nanoformulation of aminoflavone (AF) in enhancing the therapeutic index of AF in TNBC was tested [91]. More specifically, a micelle nanoparticle loaded with AF and conjugated with GE11, a peptide containing 12 amino acids, was evaluated in targeting epidermal growth factor receptor. Addition of the GE11 targeting peptide led to upgraded cellular uptake and significant growth inhibitory effects in TNBC cells. Therefore, it was suggested that AF-loaded, EGFR-targeted micelle nanoparticles constitute a promising therapeutic option for EGFR over-expressing in TNBC.

In 2017, Wali et al. conducted a study in which 128 investigational drugs as either single agents or in 768 pairwise drug combinations were evaluated as potential treatments in TNBC [92]. As the results of this study indicated, combination therapies that proved to be immediately tractable to translation included ABT-263/crizotinib, ABT-263/paclitaxel, paclitaxel/JQ1, ABT-263/XL184 and paclitaxel/nutlin-3. Crizotinib is a ROS1 inhibitor. All of them presented synergistic anti-proliferative and apoptotic activity in TNBC cells. The experimental results suggest that several combination treatments are quite promising in TNBC.

It has been reported that inhibition of proteasome, a proteolytic complex associated with the degradation of ubiquitinated proteins, has been employed as a powerful treatment therapy of cell malignancy [93]. In 2018, Rinnerthaler et al. conducted a phase I and II clinical trial in which patients with metastatic TNBC, who had already been treated with at least one prior line of chemotherapy, were treated with ixazomib combined with carboplatin on days one, eight, and 15 in a 28-day cycle [94]. Based on the clinical findings, an ixazomib and carboplatin combination proved to be an effective treatment in patients with TNBC.

Bromodomain and extraterminal domain, also known as BET are proteins that regulate gene expression and are involved in cancer development [95]. Over the last years, several BET inhibitors have been developed and tested as therapeutic agents in BC [96,97]. In 2019, Park et al. presented a study in which potential anti-tumor effects of the BET inhibitor JQ1 against AR-positive TNBC cell lines were investigated [98]. To reveal the mechanisms of JQ1 effects, multiplex gene expression analysis and immunoblotting assays were used. During this study, in vivo effects of JQ1 in a xenograft model presented TNBC was examined. JQ1 provided anti-proliferative activity, inducing apoptosis and cell cycle arrest. In addition, JQ1 showed notable anticancer activity in vivo in TNBC xenograft mouse models. As a result, the BET inhibitor JQ1 is a promising therapeutic agent that should be further investigated for the treatment of TNBC.

The intramembrane-cleaving protease γ-secretase constitutes a therapeutic target for a variety of diseases [99,100]. A range of oral γ-secretase inhibitors (GSIs) have been developed and tested in humans [101]. These γ-secretase inhibitors block notch signaling and exert anti-tumor activity. In 2020, Sardesai et al. reported a phase I study, in which an oral selective gamma secretase inhibitor RO4929097 in combination with neoadjuvant chemotherapy for TNBC was evaluated [102]. The first objective was the determination of the maximum tolerated dose of RO4929097. Patients treated with carboplatin administered intravenously on day 1, paclitaxel at 80 mg weekly and RO4929097 at 10 mg daily given orally on days 1–3, 8–10 and 15–17 for six 21-day cycles. Furthermore, the dose of RO4929097 was escalated to 10 mg. Increased doses produced toxicity. Thus, 10 mg is considered to be the suitable dose level for further investigation.

Finally, in 2021, Brufsky et al. tested the combination of cobimetinib, which is an inhibitor of the MAPK pathway, with chemotherapy [103]. It inhibits the MEK1 and MEK2 proteins, which play a vital role in the cell cycle, especially in proliferation. In this regimen, they co-administrate atezolizumab in a subgroup of patients. All patients had locally advanced or metastatic TNBC. No increase in survival was noticed in any regimen.

### 3.6. Immunotherapy

TNBC is an aggressive subtype of cancer, incapable of attracting anti-cancer and hormone drugs due to the lack of correspondent proteins. As a result, patients diagnosed with this disease have to rely mainly on chemotherapy. In recent years, another way of treatment, immunotherapy, has gained attention, as a developing option, to treat TNBC [104].

**Figure 6:**
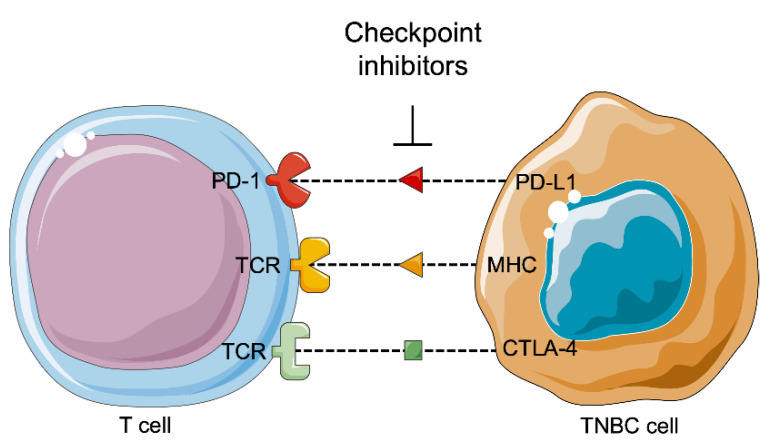
Immunotherapy against triple-negative breast cancer. PD-1: Programed cell death 1; PD-L1: Programmed cell death ligand 1; TCR: T cell receptor; MHC: Major histocompatibility complex; CTLA-4: Cytotoxic T-lymphocyte associated protein 4; TNBC: Triple negative breast cancer.

It is studied and reported that tumors can be controlled by the immune system (Figure 6). Tumor development depends on the host immune system according three phases: the elimination, equilibrium, and escape phases. The immune balance is first tilted towards anti-tumor immunity during the elimination phase, and an efficient immune system detects and then destroys the developing tumor. Several tumor cells may survive this phase and pass to the equilibrium phase, where the balance lies between anti-tumor and tumor-promoting factors, leading to a functionally suppressed state of the tumor. At the end, the tumor cells obtain the ability to call off immune surveillance and destruction, establishing an immunosuppressive tumor microenvironment in the escape phase [105,106].

According to various studies, there is a correlation between the presence of tumor-associated macrophages and prognosis in human cancers. Experimental results revealed that macrophages can be stimulated to tumor cells, providing a therapeutic approach for multiple clinical trials in cancer. In addition to macrophages, other immune-regulatory receptors could also play a complementary role in immunotherapy of cancer [104]. Neutrophils, mast cells, myeloid-derived suppressor cells, dendritic cells, natural killer cells, and adaptive immune cells (T and B lymphocytes) are some of the immune-regulatory receptors that play a significant role in immunotherapy of cancer [107].

In 2016, Nanda et al. reported a study in which the antitumor activity of the programmed cell death protein 1 (PD-1) inhibitor pembrolizumab in patients with TNBC was investigated [108]. Among 111 patients with TNBC 58.6% had PD-L1-positive tumors. Among the 27 patients who were enrolled and tested for antitumor activity, the overall response rate was 18.5%, the median time to response was 17.9 weeks and the median duration of response was not yet reached. It was also reported that clinical activity and efficiency of pembrolizumab was given every 2 weeks to patients with pre-treated, advanced TNBC.

A year later, Tolaney et al., conducted a phase II study in order to evaluate cabozantinib, a multikinase inhibitor, in patients with TNBC [109]. Patients received cabozantinib 60mg/day on a 3-week cycle and were treated with this therapy again after 6 weeks and then every 9 weeks. The first endpoint was objective response rate. Of 35 patients who underwent the therapy, three achieved a partial response and nine patients achieved stable disease for at least 15 weeks. The toxicities observed were fatigue, diarrhea, mucositis, and palmar–plantar erythrodysesthesia. On the basis of these data, cabozantinib showed efficacy signals but did not meet the primary endpoint.

In 2018, Schmid et al. conducted a phase III trial in which patients with untreated metastatic TNBC were randomly assigned to receive atezolizumab plus nab-paclitaxel or placebo plus nab-paclitaxel [110]. In each group 451 patients participated. The median progression-free survival was 7.2 months for patients who treated with atezolizumab plus nab-paclitaxel, compared with 5.5 months for patients who were treated with placebo plus nab-paclitaxel. Among patients with PD-L1-positive tumors, the median progression- free survival was 7.5 months and 5 months, respectively. Moreover, the median overall survival was 21.3 months for patients who received atezolizumab plus nab-paclitaxel and 17.6 months for patients who received placebo plus nab-paclitaxel. Additionally, among patients with PD-L1-positive tumors, the median overall survival was 25 months and 15.5 months, respectively. As a result, atezolizumab plus nab-paclitaxel provided progression-free survival among patients suffering of TNBC.

Except for PD-L1 inhibitors, a study was also conducted for CTLA-4 blockade [111]. CTLA-4 is a transmembrane receptor of T cells, which binds to B7 segment of the T cells in order to down-regulate their immune response against cancer cells. CTLA-4 is over-expressed in TNBC cells. CTLA-4 immunotherapy exerted synergistic action with DZ- 2384, which is a microtubule-targeting agent. In preclinical models, this combination was superior and with fewer side-effects, comparing to CTLA-4 immunotherapy and taxanes.

A pilot study was conducted in 2018, which examined the combination of CTLA-4 and PDL-1 inhibition in 18 patients with advanced BC, hormone positive or TNBC [112]. The most common side-effects were rash, hepatitis, and electrocyte abnormalities. This combination was more effective in patients with TNBC, as it increased cytotoxicity of T-cells and lead to clonal T-cell expression. Responses were made only in patients with TNBC (ORR = 43%), who had higher mutational gene expression and up-regulation of perforin 1 and CD8.

In the following year, Cortés et al. conducted a phase III trial in which the PD-L1 inhibitor atezolizumab was evaluated as treatment for PD-L1-positive metastatic TNBC [113]. Combining atezolizumab with first-line nab-paclitaxel provided significant improvement in progression-free survival and had a notable clinically effect on overall survival concerning patients with PD-L1-positive tumors. Moreover, patients were randomized to be treated with atezolizumab 1200 mg or placebo every 3 weeks with the chosen chemotherapy, continued until progression, showing unacceptable toxicity or withdrawal.

Voorwerk et al. conducted a phase II clinical trial, in which they examined ways to enhance sensitivity of PD-L1 blockade [114]. Sixty-seven patients were randomized to nivolumab only or radiation, cyclophosphamide, cisplatin or doxorubicin followed by nivolumab. The most effective responses were done in the doxorubicin and cisplatin groups with ORR 35% and 23% respectively. After the use of this chemotherapeutic regimens, up-regulation of PD-L1 pathway and increase in inflammation and T-cell cytotoxicity occurred. Thus, the administration of these drugs before immunotherapy might enhance its action.

Recently, Winer et al. compared the use of pembrolizumab and chemotherapy in patients with metastatic TNBC [115]. Pembrolizumab did not increase survival rates and showed various adverse effects. These findings showed that monotherapy with pembrolizumab is not more effective than chemotherapy in this type of cancer.

Table 1 summarizes all the aforementioned studies regarding investigational drug treatments for TNBC.

## 4. Discussion

TNBC remains an aggressive subtype of BC with poor prognosis. It occurs in younger women and constitutes an uncommon subtype of BC [7]. Targeted therapies gain attention and constitute a promising and developing therapeutic tool for TNBC.

The mTOR inhibitors have been studied in various trials [25,26,27,28,29,30]. PI3kb/mTOR inhibitors, such as temsirolimus, everolimus, and AZD8186 presented improved results during the experimental studies. They were co-administered with anti-angiogenetic factors, conventional chemotherapy, and other regimens.

It should be mentioned that caloric restriction exerts an influence on the mTOR pathway, and probably on metastatic TNBC [116]. The expression of PI3K aberrations, seems a positive prognostic factor for better response to the treatment [26]. Thus, tissue examination before treatment might provide useful information for patients, who can benefit from this type of treatment.

PARP inhibitors are also tested for possible anti-tumor effect against TNBC [39,40,41,42,43,44]. The results seem controversial. PARP inhibitors, such as iniparib and veliparib, did not lead to meaningful results, while tolazoparib presented potential antitumor activity. Some studies show no benefit or increased toxicity [39,41,43], whereas others demonstrated significant clinical response and improvement [42,44]. Squamous cell carcinomas were reported as a side-effect.

Aurora kinase inhibitors, when combined with taxanes, showed an anti-tumor efficacy [58]. Alisetib can be used in order to optimize the combination therapies, but AMG 900 failed to be beneficial. It should be noted that the expression of p-53 in cancer tissue improves response to the treatment [59]. However, they also have side-effects, such as hematological disorders, diarrhea, and rashes, rendering the conduction of more studies a necessity.

HDACIs are also a promising therapeutic intervention against TNBC. SAHA managed to inhibit effectively the growth of TNBC cells. The combinations of OBP-801 with eribulin and TSA with doxorubicin led to promising therapeutic strategies, through synergistic action with other agents such as olaparib, entinostat, and eribulin [65,66,67,68,69]. They induce apoptosis and inhibit angiogenesis.

Among other inhibitors, selinexor, a nucleo-cytoplasmic transport inhibitor, presented potential therapeutic activity [75]. Additionally, CRLX101, an investigational nano-particle drug, also showed anti-angiogenic therapy for TNBC. A promising therapeutic option for EGFR over-expression in TNBC proved to be a nanoformulation of aminoflavone [91]. Moreover, ixazomib, a proteasome inhibitor proved to be an effective option for TNBC treatment, when used with chemotherapy regimens [94]. Finally, a BET inhibitor, JQ1 proved to be quite beneficial and should be further investigated as a treatment tool for TNBC [98], through the induction of cell cycle arrest and apoptosis.

Immunotherapy, being a developing treatment option for patients with TNBC, resulted in enhanced outcomes [108,109,110,111,112,113,114,115]. Immunotherapy leads to recognition of cancer cells from the immune system. The expression of PDL-1 from the cancer cells shows possible better response to treatment with pembrolizumab or atezolizumab. The most beneficial anticancer activity was observed when atezolizumab was tested providing progression-free survival among patients suffering of TNBC [113], whereas pembrolizumab was not that effective [115]. These were safe drugs with few side-effects, mainly gastrointestinal ones. It should be noted that immunotherapy seems more effective when combined with chemotherapy. Finally, various clinical trials are now taking place checking the synergistic action of PARP inhibitors with immunotherapy (NCT02657889, NCT03330405) [117].

## 5. Conclusions

In conclusion, TNBC is associated with bad clinical outcomes. As a result, targeted therapies for TNBC have attracted researchers’ attention, in order for new therapeutic tools to be developed. To date there are no efficient targeted therapies for TNBC, with surgery, radiotherapy, and chemotherapy the primary reliable therapeutic options. Consequently, it is crucial that significant research is carried out in order for other molecular targeted therapies to be developed. The deeper understanding of the biological mechanism that leads to TNBC progression is improving and may result in the development of new anticancer therapies. Moreover, pre-clinical evidence of notable interactions between signaling pathways should be taken into consideration and more clinical trials should be conducted in order not only to examine new targeted drug development, but also for the development of combination of drugs with therapeutic value for patients with TNBC. Thus, the targeted therapies will offer personalized medicine with better response to treatment and fewer side-effects.

## Figures and Tables

**Table 1:** Clinical trials with targeted therapies conducted in patients with triple-negative breast cancer (TNBC).

Study			Sample Source	Therapy	Pathway/Mechanism of Action	Results
1	Ganesan et al., 2014 [25]	Phase I trial.	106 (98 evaluated) consecutive patients with advanced or metastatic TNBC.	Chemotherapy only (n = 8), combination chemotherapy and targeted therapy (n = 62), single-agent targeted therapy (n = 16), and targeted therapy with 2 or more agents (n = 20).	PI3K/AKT/mTOR.	Treatment with anti-angiogenic factors and/or PI3K/AKT/mTOR inhibitors demonstrated prolonged free survival in patients with metastatic TNBC respectively (*p* = 0.023 and *p* = 0.018).
2	Huck et al., 2014 [57]	In vivo study in immunocompromised mice-followed by clinical study.	In vivo models of TNBC grown in immune compromised mice.	60 and 80 mg/m^2^ of paclitaxel (every week), MLN8237 twice a day.	Aurora kinase inhibitor.	The highest dose of MLN8237 and paclitaxel offer the best efficacy.
3	Llombart-Cussacet al., 2015 [39]	Phase II trial.	141 patients with TNBC Stage II-IIIa.	Praclitaxel (80 mg/m^2^, n = 47) alone or in combination with iniparib, either once weekly (11.2 mg/kg, n = 46) or twice weekly (5.6 mg/kg, n = 48) for 12 weeks.	PARP inhibitor.	Best overall response in the breast (60, 61 and 63%) and breast conservation rate (53, 54 and 50%). Addition of iniparib to weekly praclitaxel did not add relevant antitumor activity or toxicity.
4	Min et al., 2015 [65]	In vitro and in vivo studies.	TNBC cell lines, xenografts models.	SAHA in combination with olaparib.	HDACIs and PARP inhibitors.	Down-regulation of the proliferative signaling pathway, increased apoptotic and autophagic cell death, and accumulation of DNA damage.
5	Arango et al., 2015 [75]	In vitro and in vivo cell lines.	26 TNBC patient-derived xenografts (PDXs).	Selinexor was combined with paclitaxel, carboplatin, eribulin, gemcitabine and doxorubicin.	Nucleo-cytoplasmatic transport inhibitor.	Selinexor as a single agent reduced tumor growth in vivo in 4 of 5 different TNBCPDX models, with a median tumor growth inhibition ratio of 42% and demonstrated greater antitumor efficacy in combination with paclitaxel or eribulin.
6	Mitri et al., 2015 [81]	Phase I study.	9 patients with TNBC.	Escalating doses of dinaciclib given on day 1 followed by standard dose of epirubicin given on day 2 of a 21-day cycle.	Cyclin dependent kinase inhibitor.	Dose escalation did not proceed past the second cohort due to toxicity. The first dose level was also found to be too toxic. No treatment responses were noted, median time to progression was 5.5 weeks.
7	Tolaney et al., 2015 [83]	Phase II study.	22 patients with TNBC.	Twice daily oral dosing of tivantinib (360 mg po bid) during a 21-day cycle.	Tyrosine kinase inhibitor.	The overall response rate was 5% (95% CI 0–25%) and the 6-month PFS was 5% (95% CI 0–25%), with 1 patient achieving a partial response.
8	Basho et al., 2016 [26]	Phase I trial.	52 women with metaplastic TNBC.	Liposomal doxorubicin, bevacizumab and temsirolimus (DAT) (n = 39) or liposomal doxorubicin, bevacizumab, and everolimus (DAE) (n = 13).	PI3K/AKT/mTOR.	The response rate was 21% (complete response = 4, 8%, partial response = 7, 13%) and 19% of patients had stable disease for at least 6 months, for a clinical benefit rate of 40%.
9	Kummar et al., 2016 [41]	Phase II study.	45 adult patients with TNBC.	Oral cyclophosphamide 50 mg once daily with or without oral veliparib at 60 mg daily in 21-day cycles.	PARP inhibitors.	Response rates and median PFS did not significantly differ between the 2 groups. The addition of veliparib to cyclophosphamide, did not improve the response rate.
10	Pham et al., 2016 [86]	In vivo preclinical study with xenografts.	Preclinical mouse models of orthotopic primary TNBC xenografts.	Bevacizumab and CRLX101.	Anti-VEGF.	CRLX101 showed antitumor efficacy, reduced metastasis, and prolonged survival.
11	Brinkman et al., 2016 [91]	In vivo study in mice.	Human TNBC cell lines.	Aminoflavone 7 mg/kg intravenously every 4 days.	Anti-EGFR.	Aminoflavone demonstrated antitumor efficacy against EGFR- over-expressing TNBC.
12	Nanda et al., 2016 [108]	Phase I clinical trial.	111 patients with TNBC.	Pembrolizumab given intravenously at 10 mg/kg every 2 weeks.	Anti-PD-1.	The overall response rate was 18.5%, the median time to response was 17.9 weeks and the median duration of response was not reached.
13	Evans et al., 2017 [42]	Normal and tumor DNA sequencing, RNASeq, and reverse phase protein arrays (RPPA), immunohistochemistry and in vivo treatment in BC patient derived xenografts.	26 patient-derived xenografts, obtained from surgical samples of recurrent tumors from 25 patients.	Use of chemotherapy with trametinib, buparlisib and/or talazoparib.	PARP inhibitor.	Talazoparib caused dramatic regression in 5 of 12 PDXs. 4 of 5 talazoparib-sensitive models did not harbor germline BRCA1/mutations, but several had somatic alterations in homologous repair pathways, including ATM deletion and BRCA2 alterations.
14	Wali et al., 2017 [92]	Clinical study.	TNBC cell lines.	128 investigational drugs as either single agents or in 768 pairwise drug combinations.	ROS1 inhibitor.	The ABT-263/crizotinib combination offers a rapid path to clinic demonstrated RTK blockade, inhibition of mitogenic signaling and pro-apoptotic signal induction in basal and mesenchymal stem-like TNBC.
15	Tolaney et al., 2017 [109]	Phase II study.	35 patients with TNBC.	Cabozantinib (60 mg daily) on a 3-week cycle and were restaged after 6 weeks and then every 9 weeks.	Tyrosine kinase inhibitor.	3 patients achieved a partial response, 9 patients achieved stable disease for at least 15 weeks, and thus the clinical benefit rate was 34%/Median PFS was 2 months. 2 patients had TNBC with MET amplification.
16	Basho et al., 2018 [27]	Phase I trial.	43 patients with non-metaplastic TNBC and 59 patients with advanced metaplastic BC.	mTOR inhibition weekly (temsirolimus or everolimus) with liposomal doxorubicin and bevacizumab every 3 weeks (DAT/DAE).	PI3K/AKT/mTOR inhibition and anti-VEGF.	Median PFS for the non-metaplastic TNBC and MpBC patients was 2.5 months and 4.8 months, respectively. Median OS for the non-metaplastic TNBC and MpBC patients was 3.7 months and 10 months, respectively. DAT/DAE appeared to be more effective in MpBC compared with non-metaplastic TNBC.
17	Carducci et al., 2018 [58]	In-human trial included dose-escalation and dose-expansion phases.	Patients with 3 tumor types: taxane- and platinum-resistant ovarian cancer, taxane-resistant TNBC, and castration-resistant and taxane- or cisplatin/etoposide resistant prostate cancer.	AMG 900 for 4 days on/10 days off at 1–50 mg/day.	Aurora kinase inhibitors.	3 of 29 (10.3%, 95% CI:2.0–28.0%) patients with ovarian cancer showed partial response. median duration of response was 24.1 weeks (95% CI: 16.1–34.1). 7 patients (24.1%, 95% CI:10.3–43.5%) experienced partial response. 5/9 patients positive for p53 expression responded to treatment. No objective responses were observed in patients with TNBC or CRPC.
18	Ono et al., 2018 [66]	Flow cytometry analysis.	TNBC cell lines.	OBP-801 or OBP-801 in combination with eribulin.	HDACIs.	Suppression of Bcl-xL and the MAPK pathway.
19	Song et al., 2018 [67]	MTT dye reduction method.	TNBC cell lines HCC1806 and HCC38.	Trichostatin A (TSA) or TSA in combination with doxorubicin.	HDACIs.	Decreased expression of CYCLIN D1, CDK4, CDK6 and BCL-XL, but increased P21 expression and inhibition of the proliferation of HCC1806 and HCC38 cells.
20	Rinnerthaler et al., 2018 [94]	Phase I and II clinical trials.	Patients with metastatic TNBC, already treated with at least 1 prior line of chemotherapy.	Ixazomib in combination with carboplatin on days 1, 8, and 15 in a 28-day cycle. The phase I part of this study utilizes an alternate dose escalation accelerated titration design. After establishing the maximum tolerated dose, the combination will be further evaluated (phase II, including 41 evaluable patients).	Proteasome inhibitor.	The results will be recorded in the future.
21	Schmid et al., 2018 [110]	Phase III trial.	451 patients with untreated metastatic TNBC.	Atezolizumab plus nab-paclitaxel or placebo plus nab-paclitaxel.	Anti-PD-L1.	The median overall survival was 21.3 months with atezolizumab plus nab-paclitaxel and 17.6 months with placebo plus nab-paclitaxel. Among patients with PD-L1-positive tumors, the median overall survival was 25 months and 15.5 months, respectively.
22	Bernier et al., 2018 [111]	In vivo study.	Mice with TNBC	CTLA-4 inhibitor and DZ- 2384 co-administration.	CTLA-4 inhibition.	CTLA-4 immunotherapy exerted synergistic action with DZ- 2384. In preclinical models, this combination was superior and with less side-effects, comparing to CTLA-4 immunotherapy and taxanes.
23	Santa-maria et al., 2018 [112]	Pilot study	18 patients with advanced estrogen receptor positive BC or TNBC	Durvalumab and tremelimumab.	PD-1/PD-L1/CTLA-4 inhibition.	This combination was more effective in patients with TNBC, as it increased cytotoxicity of T-cells and lead to clonal T-cell expression. Responses were made only in patients with TNBC (ORR = 43%), who had higher mutational gene expression and up-regulation of perforin 1 and CD8.
24	Lee et al., 2019 [28]	Phase I trial.	Patients with metastatic TNBC.	Everolimus and eribulin in different dosages combination in 25 patients.	PI3K/AKT/mTOR inhibition.	Among the 25 patients, 9 were stable, 9 reported partial response and 7 had progressive disease. Toxicity due to chemotherapy included hematological disorders, fatigue, stomatitis and hyperglycemia.
25	Maiti et al., 2019 [68]	Sphere formation assay.	TNBC cell lines.	Entinostat.	HDACIs.	Re-expression of the anti-angiogenic genes, serpin family F member 1 (SERPINF1) and thrombospondin 2 (THBS2), and to that of the tumor suppressor genes, phosphatase and tensin homolog (PTEN) and p21, and reduced VM structures. Down-regulation of the expression of vascular endothelial growth factor A (VEGF-A), and that of the epithelial-mesenchymal transition (EMT)-related genes, Vimentin and β-catenin.
26	Park et al., 2019 [98]	In vivo study.	A xenograft model of AR expressing TNBC in mouse models.	BET inhibitor JQ1.	BET inhibitor.	JQ1 showed significant anti-tumor activity in vivo in TNBC xenograft mouse models as a monotherapy and in combination with anti-AR therapy.
27	Cortés et al., 2019 [113]	Phase III trial.	Patients with PD-L1-positive tumors.	Atezolizumab 1200 mg or placebo every 3 weeks with the chosen chemotherapy.	Anti-PD-L1.	Unacceptable toxicity or withdrawal.
28	Voorwerk et al., 2019 [114]	Phase II trial.	67 patients with TNBC.	Nivolumab only or radiation or cyclphosphamide or cisplatin or doxorubicin all followed by nivolumab.	Anti-PD-1.	The most effective responses were done in the doxorubicin and cisplatin groups with ORR 35% and 23% respectively. After the use of this chemotherapeutic regimens, up-regulation of PD-L1 pathway and increase in inflammation and T-cell cytotoxicity occurred. Thus, the administration of these drugs before immunotherapy might enhance its action.
29	Owusu-Brackett et al., 2020 [29]	In vitro cell viability assay.	TNBC cell lines.	AZD8186 in combination with paclitaxel, eribulin.	PI3K/AKT/mTOR inhibition.	AZD8186 had single agent efficacy in PTEN-deficient TNBC cell lines in vitro but had limited single agent efficacy in vivo. AZD8186 had enhanced efficacy when combined with paclitaxel and anti-PD1 in vivo.
30	Pothuri et al.,2020 [43]	Clinical trial.	44 patients with ovarian or TNBC.	Veliparib and doxorubicin in various dosages.	PARP inhibitor.	Although complete clinical response was observed in two cases, and the anti-tumor efficacy was generally acceptable, complications such as oral squamous cell carcinomas appeared.
31	Tolcher et al.,2020 [59]	Clinical trial.	126 patients with TNBC or melanoma.	Trametinib and uprosertib in various dosages.	Aurora kinase inhibitors.	The anti-tumor efficacy was minimal, whereas adverse effects such as severe diarrheas or rashes appeared.
32	Milazzo et al., 2020 [69]	In vitro and in vivo studies.	TNBC cell lines, xenografts models.	ST8176AA1 (ADC).	HDACIs.	Higher anti-tumor activity of ST8176AA1 compared to trastuzumab, increased expression of ErbB2 and estrogen receptor in TNBC cells, lower expression of the proliferation marker Ki67 and higher expression of cleaved caspase-3 in mice treated with the ADC compared to those treated with trastuzumab.
33	Sardesai et al., 2020 [102]	Phase I study.	Patients with TNBC.	Carboplatin on day 1, weekly paclitaxel at 80 mg and RO4929097 10 mg daily given orally on days 1–3, 8–10 and 15–17 for 6 21-day cycles. RO4929097 was escalated in 10 mg using the 3 + 3 dose escalation design.	γ-secretase inhibitor.	RO4929097 at 10 mg would have been the likely dose level for further development.
34	Ma et al., 2021 [30]	In vitro study.	MDA-MB-231, A549 and HeLa cell lines.	Anilide.	PI3K/AKT/mTOR inhibition.	Anilide enhance apoptosis and inhibit the migration and the proliferation of TNBC cells.
35	Eikesdal et al., 2021 [44]	Clinical trial.	32 patients with TNBC, who have not received previously chemotherapy.	Olaparib.	PARP inhibitor.	Olaparib is effective against treatment-naïve TNBC cells with HR deficiency.
36	Brufsky et al.,2021 [103]	Phase II clinical trial.	Patients with locally advanced or metastatic TNBC.	Cobimetinib plus chemotherapy, with or without atezolizumab.	MAPK inhibition.	No increase in survival was noticed in any regimen.
37	Winer et al., 2021 [115]	Clinical trial.	1098 patients with metastatic TNBC.	Pembrolizumab versus chemotherapy.	Anti-PD-1.	Pembrolizumab did not increase survival rates.
